# Hyperammonaemic encephalopathy due to non-functioning urea cycle as a complication to gastric bypass surgery

**DOI:** 10.1007/s11011-024-01434-4

**Published:** 2024-11-28

**Authors:** Kristoffer Kjærgaard, Peter Lykke Eriksen, Thomas Kromann Nøhr, Steen Bønløkke Pedersen, Claus Højbjerg Gravholt, Hendrik Vilstrup, Karen Louise Thomsen

**Affiliations:** 1 Department of Hepatology and Gastroenterology, Aarhus University Hospital, Palle Juul-Jensens Boulevard, Aarhus, Denmark; 2 Department of Clinical Medicine, Aarhus University, Aarhus, Denmark; 3 Steno Diabetes Center, Aarhus University Hospital, Aarhus, Denmark; 4 Department of Endocrinology, Aarhus University Hospital, Aarhus, Denmark; 5 Department of Molecular Medicine, Aarhus University Hospital, Aarhus, Denmark

**Keywords:** Hyperammonaemia, Gastric bypass, Encephalopathy, Urea cycle dysfunction

## Abstract

Hyperammonaemic encephalopathy in the absence of liver failure is a major diagnostic challenge. A rare cause is as a complication to previous gastric bypass surgery, a condition reported to be associated with high mortality. In this case report, we present the exhaustive diagnostic work-up and clinical reversal of deep and recurrent hyperammonaemic encephalopathy in a patient with previous gastric bypass surgery. As a key finding, the patient exhibited an extreme reduction of the in vivo capacity for urea synthesis, which was reverted by long-standing correction of severe protein and micronutrient malnourishment (Functional Hepatic Nitrogen Clearance; 2.9 to 25.5 L/h). In addition, we observed reduced levels of fasting plasma amino acids (α-amino nitrogen; 2.7 to 3.6 mmol/L) and glucagon (0.3 to 2.6 pmol/L) before clinical improvement, which may contribute to the non-functioning urea synthesis. These observations elucidate the underlying pathophysiology of hyperammonaemia as a complication of gastric bypass and highlight a potential mechanism – non-functioning urea cycle as a result of protein malnourishment and hepatic glucagon resistance.

**Supplementary Information:**

The online version contains supplementary material available at 10.1007/s11011-024-01434-4.

## Introduction

Hyperammonaemic encephalopathy in the absence of liver failure is rare and represents a major diagnostic challenge (Kalra and Norvell 2020). 95% of critical care admissions due to hyperammonaemia are caused by liver failure, whereas other causes include genetic urea cycle disorders, extensive portosystemic shunting and valproate treatment (Sakusic et al. 2018; Walker 2012; Nøhr et al. 2021). A rare but devastating cause of hyperammonaemia is gastric bypass surgery, described in several case reports with one systematic review reporting a mortality rate of 50% (Fenves et al. 2015). The condition may in some cases be explained by unmasking of inborn errors of the urea cycle or fatty acid oxidation (Singh et al. 2015). Contributory factors may include sarcopenia, micronutrient deficiency, hormonal disturbances, and disruption of the gut microbiota, which are all frequent complications of gastric bypass surgery (Adeva et al. 2012). However, the underlying pathophysiological mechanisms by which gastric bypass surgery may trigger severe cases of hyperammonaemia remain unravelled.

In this case report, we describe the exhaustive diagnostic work-up and clinical reversal of deep and recurrent hyperammonaemic encephalopathy in a patient with previous gastric bypass surgery. We propose a novel mechanism for hyperammonaemia in this condition to be severely impaired capacity for urea synthesis caused by chronic malnutrition and protein deficiency.

## Case presentation

### Clinical course

**Fig. 1:**
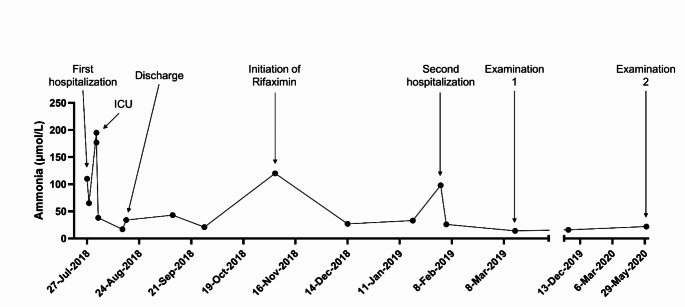
Timeline of plasma ammonia levels measured during hospitalizations, in the outpatient clinic, and at examinations. *ICU*: Intensive Care Unit

In July 2018, a 46-year-old Caucasian female was acutely admitted to the hospital after a two-hour incidence of confusion with complete amnesia and malaise, preceded by a few weeks of diarrhoea and minimal food intake. She had a medical history of morbid adiposity and Roux-en-Y gastric bypass in 2007, which led to a weight loss from 80 kg to 59 kg. She described recurrent episodes of confusion over the past five years, disabling her ability to work. Physical examination revealed pronounced response latency, impaired consciousness, and extensive peripheral oedema. Initial biochemistry showed alkaline phosphatase 333 U/L, γ-glutamyl transferase 157 U/L, INR 2.1, albumin 17 g/L, CRP 73 mg/L, and hyperammonaemia (110 µmol/L; Fig. 1), whilst bilirubin (28 µmol/L) and alanine aminotransferase (49 U/L) were just marginally elevated. Computed tomography (CT) scan of the brain and cerebrospinal fluid analysis were unremarkable. Thus, liver failure of unknown aetiology was suspected with West Haven grade 2–3 overt hepatic encephalopathy (HE). Abdominal CT scan showed gross hepatomegaly with steatosis but no signs of cirrhosis. *Campylobacter jejuni* was detected in faeces, and deficiencies in serum zinc, vitamin A, and vitamin E were noted.

Treatment with parenteral nutrition and micronutrient supplementation including intravenous vitamin K was initiated leading to the normalization of INR and reduced ammonia levels (65 µmol/L, Fig. 1) within 2 days. However, a few days later, the patient developed sudden loss of consciousness (GCS 12), hyperventilation and involuntary movements accompanied by severe hyperammonaemia (195 µmol/L), requiring transfer to the intensive care unit. The condition gradually improved by treatment with lactulose, tube-fed moderate hyperalimentation, anti-convulsive therapy, and pragmatic administration of broad-spectrum antibiotics. She was discharged from hospital after one month.

Two months later (November 2018), the patient was seen in the outpatient clinic complaining about fatigue, poor memory, and episodes of loss of consciousness. EEG was normal, whereas the psychometric test Continuous Reaction Time (CRT) (Lauridsen et al. 2012) revealed a decreased CRT index of 1.4 (diagnostic threshold for minimal HE < 1.9) along with recurrence of hyperammonaemia (120 µmol/L, Fig. 1). Rifaximin therapy was initiated with a subsequent reduction in ammonia (26 µmol/L, Fig. 1).

Half a year later (March 2019), the patient had lost another 20 kg, resulting in a body weight of 40 kg and a BMI of 14.2 kg/m^2^. By then, a comprehensive diagnostic work-up was initiated (Ex1) including a liver biopsy with histological evaluation and gene expression analysis, investigation of inborn metabolic disorders, measurement of the in vivo ureagenesis capacity (Functional Hepatic Nitrogen Clearance; FHNC), and psychometric testing. Moreover, continuous glucose monitoring (CGM) revealed recurrent episodes of hypoglycaemia, and a finding of low faecal elastase suggested an element of exocrine pancreatic insufficiency. After the diagnostic work-up, the patient was started on a nutrition regime with moderate protein and carbohydrate hyperalimentation, including supplements with branched chain amino acids (BCAA) and pancreatic enzyme replacement. Over the next year, the patient's clinical condition substantially improved, and she reached her ideal body weight of 60 kg with no oedema, but remained fatigued. Two years later (June 2020), liver biopsy, psychometric testing, and measurement of the FHNC were repeated (Ex2). Three years later (March 2021), the anti-HE treatment (rifaximin, BCAA) was discontinued, and her condition has remained stable till today with no episodes of encephalopathy or hyperammonaemia. Figure 1 illustrates the course of plasma ammonia levels over the period.

### Diagnostic work-up for causes of hyperammonaemia

The initial liver biopsy showed only mild fibrosis (F1) and dilation of the portal veins but no steatosis or inflammation. Portosystemic shunting was excluded by a contrast-enhanced CT scan of the abdomen. Investigations of non-cirrhotic causes for hyperammonaemia showed no abnormal expression of genes involved in nitrogen metabolism by RNA sequencing of liver tissue, normal urine purine and pyrimidine metabolite levels, and normal free carnitine and acetylcarnitine levels in plasma. Plasma levels of the essential amino acids citrulline and arginine were discreetly reduced, while glutamine and ornithine were normal. Thus, genetic urea cycle defects, mitochondrial fatty acid oxidation defects, and amino acid abnormalities were excluded (Laish and Ben Ari 2011).

### Urea synthesis capacity and psychometric performance before and after clinical improvement

As part of the investigations before and after clinical improvement, in vivo measurement of the urea synthesis capacity (FHNC) and psychometric testing for minimal HE (mHE) were performed. The FHNC method utilizes the urea synthesis rate in response to a constant infusion of alanine, as previously described (Glavind et al. 2016). At Ex1, the patient had reduced fasting blood α-amino acid (AAN) levels and exhibited an exceptionally low FHNC of 2.9 L/h (normal range 18–50 L/h (Lykke Eriksen et al. 2019; Vilstrup 1980, 1989), Table 1).

**Table 1:** Patient characteristics, urea synthesis and psychometric tests at examinations

	Body weight		Urea synthesis		Psychometrics		
	Weight (kg)	BMI (kg/m^2^)	t_0_ AAN (mmol/L)	FHNC (L/h)	PHES	CRT Index	CFF
Ex1	40	15.1	2.74	2.9	-3	2.4	43.1
EX2	61	23.0	3.62	25.5	-1	2.4	41.9

The two examinations were performed in March 2019 (Ex1) and after clinical improvement in June 2020 (Ex2)

PHES: Portosystemic Hepatic Encephalopathy Score (normal range, PHES > -4); CRT: Continuous Reaction Time (normal range, Index > 1.9); CFF: Critical Flicker Frequency (normal range, CFF > 39, mHE 36-43); AAN: α-amino Nitrogen; FHNC: Functional Hepatic Nitrogen Clearance (normal range, 18-50 L/h); NH_3_: Ammonia

**Fig. 2:**
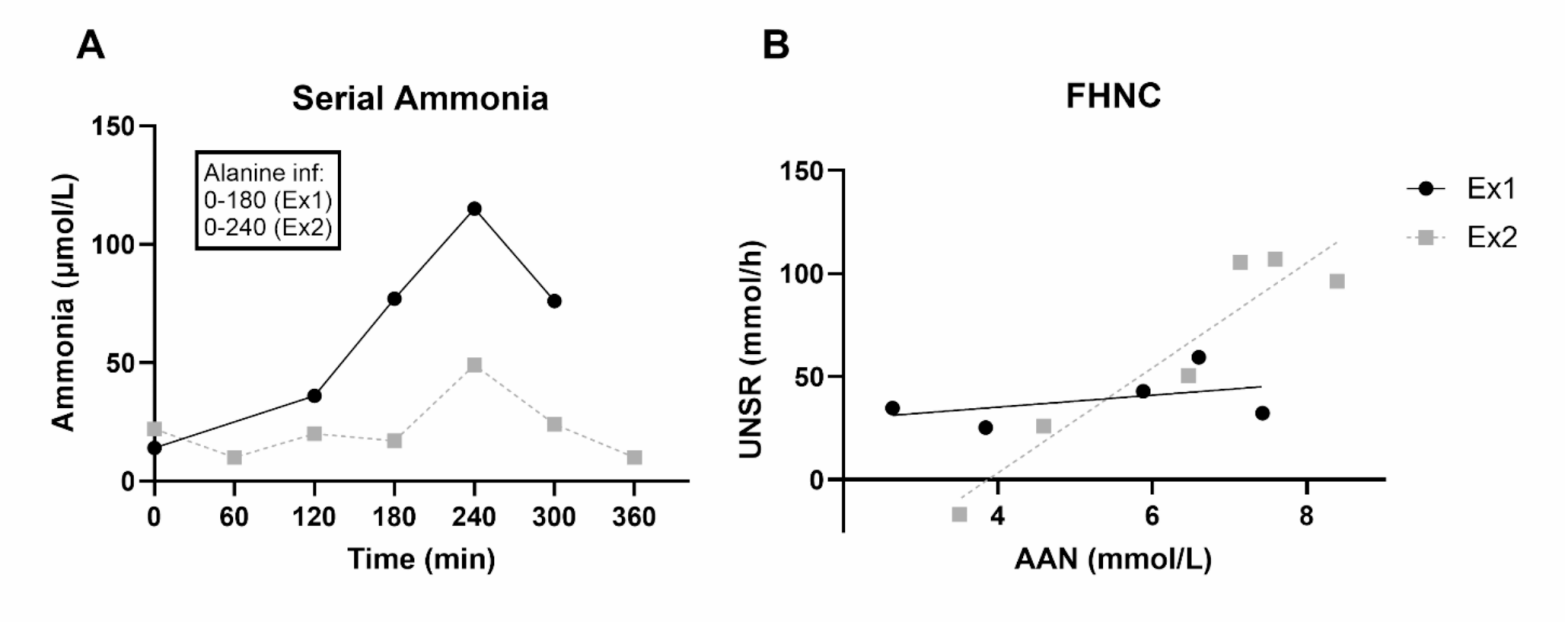
Serial ammonia measurements during alanine infusion and functional hepatic nitrogen clearance (FHNC) examination. The two examinations were performed in March 2019 (Ex1) and after clinical improvement in June 2020 (Ex2). In the FHNC investigation, urea synthesis in the liver is stimulated in the fasting state through an intravenous infusion of alanine. The infusion was initiated immediately after the first measurement (t=0) and continued for 3 (Ex1) or 4 hours (Ex2). Panel A: At Ex1, 3 hours of alanine infusion drastically increased plasma ammonia concentrations, whereas 4 hours of alanine infusion increased ammonia only marginally at Ex2. Panel B: At Ex1, the alanine infusion did almost not stimulate urea synthesis (FHNC = 2.9 L/h). At Ex2, increasing levels of α-amino acids (AAN) efficiently stimulated urea synthesis to normal levels (FHNC = 25.5 L/h). The FHNC is determined as the slope of linear regression between α-amino acids in blood and the urea nitrogen synthesis rate (UNSR), calculated as the accumulated urea in blood and urine, corrected for intestinal urea loss (Hamberg and Vilstrup 1994)

During the examination, the alanine infusion prompted an exceptionally marked rise in plasma ammonia to 115 µmol/L (Fig. 2). It should be noted that the patient did not present any cerebral symptoms during the examination. Basal glucagon was 0.3 pmol/L (95% reference range 1.5–18.0 pmol/L; Mercodia Glucagon ELISA), increasing to 3.4 pmol/L following the alanine infusion (expected basal level 6 pmol/L increasing to 22 pmol/L based on previous published data in healthy persons (Lykke Eriksen et al. 2019)). Psychometric tests, which included the Portosystemic Hepatic Encephalopathy Score (PHES) (Weissenborn et al. 2001), CRT index (Lauridsen et al. 2012), and Critical Flicker Frequency (CFF) (Sharma et al. 2007), were all within normal range (Table 1).

One year after the initiation of nutritional therapy (Ex2), the FHNC had normalised to 25.5 L/h (8-fold increase), and blood AAN increased by 30% (Table 1). Moreover, plasma ammonia did not increase during alanine infusion (Fig. 2). Basal glucagon was 2.6 pmol/L and increased to 4.1 pmol/L by alanine infusion. Psychometric results were notable for an improvement in the PHES from − 3 to -1 (both within normal range), while the CRT and CFF were unchanged (Table 1).

Between the two examinations, RNA sequencing of liver tissue showed a minor increase (10–40%) in the expression of all urea cycle enzymes (except arginase 1 [ARG1]), as well as in other genes involved in hepatic nitrogen conversion including glutamine synthetase (GS) (Eriksen et al. 2019), which was increased by 90% (Table S1). In addition, there was a marked increase in metallothionein protein expression compared to Ex1 (Table S1).

## Discussion

We present in clinical and mechanistic detail a case of recurrent deep hyperammonaemic encephalopathy linked to severe chronic malnutrition as a complication of gastric bypass surgery. As our key finding, the patient exhibited an extreme reduction of the in vivo capacity for urea synthesis, which was reverted by long-standing protein feeding in parallel with improved clinical condition. This is the first documentation of a non-functioning urea synthesis and its consequences in a patient devoid of liver failure or urea cycle disorder– the loss of urea synthesis capacity was comparable only to that observed in acute liver failure and much below that of patients with cirrhosis (Glavind et al. 2016; Vilstrup 1980, 1989). Notably, the diminished capacity for urea synthesis resulted in inadequate disposal of ammonia upon alanine infusion, which we suggest to be the cause of the recurrent invalidating encephalopathy. Our case is unique in identifying a non-functioning urea synthesis in a patient without liver disease or inborn errors of metabolism.

Previous reports of hyperammonaemia as a complication of gastric bypass surgery have attributed the condition to underlying heterozygote or low penetrance genetic errors of metabolism associated with hyperammonaemia (Singh et al. 2015). In our patient, such disorders were ruled out along with acute or chronic liver disease, portosystemic shunting, and use of medications triggering hyperammonaemia (Walker 2012). Most importantly, we found no plausible cause for the encephalopathy episodes besides hyperammonaemia. Consistent with previous cases (Singh et al. 2015), the patient was malnourished with reduced lean body mass, hypoalbuminemia, and reduced essential amino acids as well as micronutrient deficiency, all of which are well-known late complications to bariatric surgery related to intestinal malabsorption and reduced protein intake (Gletsu-Miller and Wright 2013; Tourky et al. 2022).

During the ten years after gastric bypass surgery, the patient had undergone substantial weight loss which included severe protein malnourishment leading to an anasarca-like condition. During acute presentations, the patient exhibited what mimicked acute liver failure, characterized by compromised hepatic protein synthesis with decreased coagulation factors, albumin, and even failing gluconeogenesis. The gene expression of urea cycle-related enzymes was largely intact, so the non-functioning urea synthesis may be ascribed to a post-transcriptional lack of enzyme protein synthesis. However, it was not possible to confirm this through protein analyses due to a limited quantity of liver tissue.

Urea synthesis is of essential importance to body integrity and health by regulating organ and whole-body nitrogen balance as well as eliminating toxic ammonia. The urea synthesis rate is controlled by amino acids in blood and this relationship is regulated by diet protein, interleukins and endogenous hormones, most importantly induced by glucagon (Vilstrup et al. 1990). Our patient exhibited a physiological increase in plasma glucagon in response to alanine infusion but with no corresponding increase in ureagenesis. This corresponds to findings from rodent studies showing that chronic protein starvation reduces in vivo ureagenesis, possibly by the induction of a post-receptor hepatic glucagon resistance (Petersen et al. 1990; Rao and Harsha 1995; Lizarraga-Mollinedo et al. 2011). At the same time, fasting glucagon was severely reduced along with low levels of amino acid nitrogen in blood. These observations may be interpreted as a phenomenon of non-functioning urea synthesis due to protein malnutrition and severe hypoglucagonemia, as previously reported during chronic starvation (Robinson and Seakins 1982). Glucagon exerts its ureagenic effects by both acute and chronic regulatory mechanisms, where the latter includes increased expression of urea cycle enzymes (Snodgrass et al. 1978; Richter et al. 2022). Noteworthy, also hepatic GS expression was doubled upon restoration of nutritional status, perhaps influenced by increasing glucagon as it has been shown to control GS gene expression (Cheng et al. 2018).

The patient experienced remarkable clinical improvement following targeted nutritional intervention along with a 30% expansion of the total plasma amino acid pool (AAN) and complete restoration of urea synthesis function. The treatment included pancreatic enzyme substitution, a potential key intervention, as pancreatic exocrine insufficiency is a common sequela to bariatric surgery and a mediator of intestinal malabsorption (Vujasinovic et al. 2017). Another possible contributory factor was the substitution of micronutrients, most notably zinc– an important co-factor of the urea cycle enzyme ornithine transcarbamylase, which may undergo reversible functional impairment during zinc deficiency (Marchesini et al. 1996). In support of this was the severe reduction in hepatic gene expression of metallothionein proteins, which are crucial for zinc homeostasis (Krężel and Maret 2017). Also, the reversal of sarcopenia may have contributed to increased resilience against hyperammonaemia, since skeletal muscle constitutes an important first line scavenging pathway for ammonia (Jindal and Jagdish 2019). One final possible contributor to hyperammonaemia in patients with previous gastric bypass surgery is increased ammonia formation due to an altered gut microbiota (Sabate et al. 2017), but this remains to be investigated.

**Fig. 3:**
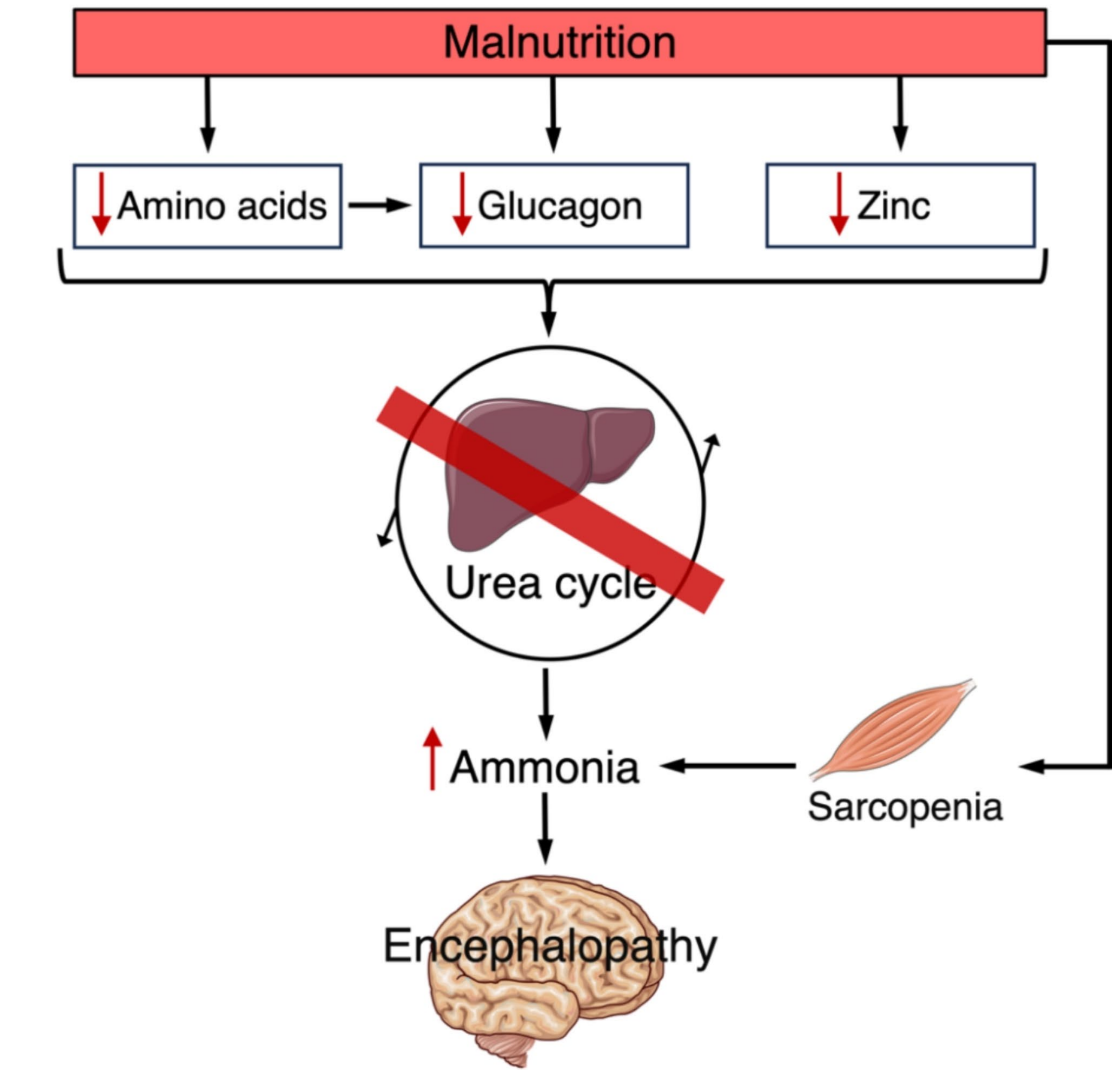
Mechanism behind malnutrition-related urea cycle dysfunction as the basis for hyperammonaemia and encephalopathy

Hyperammonaemic encephalopathy in patients without cirrhosis often necessitates intensive care, with empirical evidence suggesting a crucial role of rapid diagnosis and supportive treatment for a good prognosis (Laish and Ben Ari 2011; Walker 2012; Sakusic et al. 2018). Prior cases, as well as this one, stress the imperative need for correction of malnutrition and its precipitating factors in this patient group (Singh et al. 2015). The observations reported in the case elucidate the underlying pathophysiology of hyperammonaemia as a complication of gastric bypass and highlight a potential mechanism– non-functioning urea synthesis because of protein malnourishment and insufficient glucagon production or signalling (Fig. 3).

As such, the chronic macro- and micronutrient malnutrition that may arise as a complication to gastric bypass surgery comprises the perfect storm for hyperammonaemia with a possible fatal outcome.

## Electronic supplementary material

Below is the link to the electronic supplementary material.

Supplementary Material 1

## Data Availability

No datasets were generated or analysed during the current study.
