# Supplementary material for: Hyperammonaemic encephalopathy due to non-functioning urea cycle as a complication to gastric bypass surgery
Source: Metab Brain Dis. 2024 Nov 28;40(1):46. doi: 10.1007/s11011-024-01434-4 (PMC11604766; doi:10.1007/s11011-024-01434-4)
Supplement: Supplementary file 1 — Supplementary Material 1 [file 11011_2024_1434_MOESM1_ESM.docx]

**Table S1: Liver gene expression levels from the two investigations.**

| **Gene** | **Ex1** | **Ex2** | **Ratio (fold change) (integer)** |
| --- | --- | --- | --- |
| *Urea synthesis and nitrogen conversion* | | | |
| ARG1 | 25104 | 25612 | 1.0 |
| ASL | 6686 | 7374 | 1.1 |
| ASS1 | 38928 | 51465 | 1.3 |
| CPS1 | 94234 | 108397 | 1.2 |
| OTC | 7152 | 9167 | 1.3 |
| GLS2 | 3638 | 4617 | 1.3 |
| GLUD1 | 25698 | 35939 | 1.4 |
| GS | 15141 | 29213 | 1.9 |
| NAGS | 920 | 844 | 0.9 |
| SLC25A22 | 631 | 652 | 1.0 |
| SLC38A2 | 4749 | 4356 | 0.9 |
| SLC38A3 | 15915 | 22213 | 1.4 |
| *Metallothioneins* | | | |
| MT1H | 13 | 777 | 59.6 |
| MT1F | 224 | 2484 | 11.1 |
| MT1M | 52 | 511 | 9.8 |
| MT1G | 963 | 8530 | 8.9 |
| MT1E | 1815 | 5907 | 3.3 |
| MT2A | 3601 | 10316 | 2.9 |

*The two investigations were performed in March 2019 (Ex1) and after clinical improvement in June 2020 (Ex2). Gene expression levels (read counts) were analysed using RNA sequencing of liver tissue. ARG1: arginase 1, ASL: argininosuccinate lyase, ASS1: argininosuccinate synthetase 1, CPS1: carbamoyl phosphate synthetase 1, OTC: ornithine transcarbamylase, GLS2: glutaminase 2, GLUD1: glutamate dehydrogenase 1, GS: glutamine synthetase, NAGS: N‐acetylglutamate synthase, SLC25A22: solute carrier family 25 member 22, SLC38A2: solute carrier family 38 member 2, SLC38A3: solute carrier family 38 member 3, MT: metallothionein.*
